# Enhanced glucose utilization of skeletal muscle after 4 weeks of intermittent hypoxia in a mouse model of type 2 diabetes

**DOI:** 10.1371/journal.pone.0296815

**Published:** 2024-01-25

**Authors:** Yuqi Zhao, Chaoqun Li, Shi Zhou, Youyu He, Yun Wang, Yuan Zhang, Li Wen

**Affiliations:** 1 School of Social Sports and Health Sciences, Tianjin University of Sport, Tianjin, China; 2 School of Exercise and Health, Nanjing Sport Institute, Nanjing, Jiangsu, China; 3 School of Kinesiology, Shanghai University of Sport, Shanghai, China; 4 Faculty of Health, Southern Cross University, Lismore, Australia; Bangor University, UNITED KINGDOM

## Abstract

**Background:**

Intermittent hypoxia intervention (IHI) has been shown to reduces blood glucose and improves insulin resistance in type 2 diabetes (T2D) and has been suggested as a complementary or alternative intervention to exercise for individuals with limited mobility. Previous research on IHI has assessed cellular glucose uptake rather than utilization. The purpose of this study was to determine the effect of a 4-week IHI, with or without an aerobic exercise, on skeletal muscle glucose utilization as indicated by the changes in pyruvate, lactate, NAD^+^, and NADH, using a mouse model of diet-induced T2D. In addition, the effects of one exposure to hypoxia (acute) and of a 4-week IHI (chronic) were compared to explore their relationship.

**Methods:**

C57BL/6J mice were randomly assigned to normal control and high-fat-diet groups, and the mice that developed diet-induced diabetes were assigned to diabetes control, and intervention groups with 1 hour (acute) or 4 weeks (1 hour/day, 6 days/week) exposure to a hypoxic envrionment (0.15 FiO_2_), exercise (treadmill run) in normoxia, and exercise in hypoxia, respectively, with N = 7 in each group. The effects of the interventions on concentrations of fasting blood glucose, muscle glucose, GLUT4, lactate, pyruvate, nicotinamide adenine dinucleotide (NAD^+^), and NADH were measured, and statistically compared between the groups.

**Results:**

Compared with diabetes control group, the mice treated in the hypoxic environment for 4 weeks showed a significantly higher pyruvate levels and lower lactate/pyruvate ratios in the quadriceps muscle, and the mice exposed to hypoxia without or with aerobic exercise for either for 4 weeks or just 1 hour showed higher NAD^+^ levels and lower NADH/NAD^+^ ratios.

**Conclusions:**

Exposure to moderate hypoxia for either one bout or 4 weeks significantly increased the body’s mitochondrial NAD cyclethe in diabetic mice even in the absence of aerobic exercise. The hypoxia and exercise interventions exhibited synergistic effects on glycolysis. These findings provide mechanistic insights into the effects of IHI in respect of the management of hyperglycemia.

## 1 Introduction

Exercise is an effective intervention for the management of hyperglycemia, insulin resistance, type 2 diabetes (T2D) and their associated risk factors [1, 2]. Obtaining benefits from exercise typically requires sustained participation at a prescribed frequency, intensity, time, and type of exercise for several months or even years [3–6]. However, approximately 80% of patients with T2D are overweight or obese, and many people have limited mobility, cardiovascular disease, or other complications [7, 8] that may limit their ability to participate in regular exercise. In addition, a significant change in lifestyle is a challenge for some individuals [9]. Therefore, alternative or complementary interventions are required for some individuals in addition to pharmaceutical treatments or to help them in transition to a more physically active lifestyle [10].

Among the alternative or complementary intervention strategies, intermittent hypoxia intervention (IHI) has shown beneficial effects and is increasingly being given attention by researchers, practitioners, and patients [2]. IHI refers to the practice of repeated episodes of breathing air in which the partial pressure of oxygen is lower than normal for a certain period of time (e.g., several minutes to hours) [11] that results in a decreased partial pressure of oxygen in blood or tissues, interrupted by breathing normal air between the episodes [12]. IHI can be used in combination with exercise [13] or alone [13, 14].

The physiological mechanisms underlying improved blood glucose management in response to IHI is unclear. It has been reported that after 4 weeks of IHI, the translocation of glucose transporter type 4 (GLUT4) in skeletal muscle was increased [13]. For cells with impaired glucose uptake, the cellular dynamic balance between substrates availability and utilization (energy metabolism) has to be adjusted from the normal level [15]. At present, much of the research on the potential mechanisms underlying the effects of hypoxia, exercise, or a combination of both interventions focuses on the improvement of insulin-stimulated glucose uptake [16]. However, less attention is being paid to the utilization of the increased cellular glucose in response to these interventions. In insulin-resistant skeletal muscle, glycogen synthesis is impaired [17], as well as the mitochondrial oxidation capacity individuals with diabetes [18]. A question can be raised in this regard, whether and how the cells in individuals with diabetes have the capacity to accommodate the increased cellular glucose level.

Improvements in health in response to a chronic intervention are based on adaptation to repeated stimuli [19]. When the body is exposed to an unfamiliar stimulus, the body’s homeostasis is disturbed, causing physical stress [20–22]. Long-term repeated exposure to a stimulus induces adaptive changes so that the organism can gradually readjust the response [23–26]. Therefore, the effects of long-term IHI or exercise vs. a single exposure to a stimulus on body functions may differ. However, to date, there is a paucity of research examining the responses of the body to a single bout of hypoxia compare to the adaptations to chronic IHI, with respect to muscle glucose metabolism in the same cohort of participants within a single study.

Previous reports have demonstrated that an IHI can reduce the fasting blood glucose (FBG) and increase glucose uptake by skeletal muscle in T2D mice [13]; however, it is unclear whether the skeletal muscle can effectively utilize the increased level of glucose. Therefore, the overall purpose of this study was to determine the effect of a 4-week IHI, with or without an aerobic exercise, on skeletal muscle glucose utilization as indicated by the changes in pyruvate, lactate, NAD^+^, and NADH, using a mouse model of diet-induced T2D [13]. In addition, the effects of one exposure to hypoxic environment (acute) and of a 4-week IHI (chronic) were compared to explore their relationship.

## 2 Materials and methods

The research design and protocol were approved by the Animal Care and Ethics Committee of Southern Cross University (approval number ARA13/04).

### 2.1 Animal model of T2D and interventions

This study used a randomized controlled trial design. Ten-week-old male C57BL/6J mice, with a mean body weight of 17.5 ± 1.77 g, were obtained from a commercial provider (Beijing HFK Bioscience Co. Ltd., China). The animals were housed in the environment with temperature of 20–25°C, relative humidity of 30%-40%, and lights on for 12 hours daily. The mice had free access to water and food. After a week of adaptive feeding with a normal diet, the mice were randomly assigned to either a normal control cohort (NC) that was fed a typical rodent diet (14% protein, 72% carbohydrates, and 4% fat; the fat source was vegetable oil), or to a high-fat-diet cohort that was fed a high-fat diet (14% protein, 21% carbohydrates, and 55% fat; the fat source was lard), continuously during the experimental period.

All mice were measured for their body weight and FBG level (after a 6-hour fasting) level every two weeks. After feeding with the high-fat diet for 10–12 weeks, mice were considered to have acquired T2D when their FBG level reached 13 mmol/L [27] and hyperinsulinemia occurred at the same time [13]. Mice that developed T2D had a mean FBG level of 13.9 ± 0.69 mmol/L and were randomly placed in one of the seven groups: sedentary in normoxic environment (diabetic control; DC); sedentary for 4 weeks in hypoxic environment (DH), exercise for 4 weeks in normoxic environment (DE), exercise for 4 weeks in hypoxic environment (DHE), acute (one bout) sedentary in hypoxic environment (DH1), acute exercise bout in normoxic environment (DE1), and acute exercise bout in hypoxic environment (DHE1). Together with the NC, a total of 56 mice were used, with seven mice in each group. The NC and DC groups were used as the control for both the acute intervention and the 4-week intervention to minimize the number of animals used in the study. All mice were sampled at around 26 weeks of age after the intervention period.

### 2.2 Intervention protocol

The mice in the hypoxia intervention groups were placed in a hypoxia tent (Don Whitley H35 workstation, U.S.A.) with the fraction of oxygen in the air maintained at 0.150 ± 0.0046 (15.0 ± 0.46%) for 60 min on each treatment day [28]. The hypoxia tent is large enough to fit the animal treadmill and a researcher inside. The oxygen concentration in the tent was continuously monitored and maintained at the required level. Intermittent hypoxia in a broad sense refers to the reciprocation of hypoxic and normoxic conditions at certain intervals [29]. In this study, the “intermittent” referred to one hour (continuous) exposure to a hypoxic environment followed by 23 hours in a normoxic environment on each intervention day. The hypoxia condition is regarded as moderate, under which skeletal muscles have shown adaptations [30–32].

The mice in the exercise groups ran on a custom-built motor-driven animal treadmill at a slope of 0°. In each exercise session the initial speed was 10 cm/s for 12 min, then the speed was increased by 2 cm/s every 12 min, for a total of 60 min in each exercise session. The exercise intensities were estimated as in a range of 75% to 90% of the maximal oxygen consumption for mice [33]. Although the actual oxygen consumption rate was not monitored in this study, the exercise can be regarded as predominantly aerobic in nature because it was a continuous exercise with incremental workload for 60 min that was well tolerated by the mice [34].

During each intervention session, a researcher was in the hypoxia tent to control the exercise protocol, and monitor the oxygen level using a portable monitor and the responses of the mice. The chronic intervention groups received six one-hour treatment sessions per week for 4 weeks [35]. The interventions were delivered during the day time (8 am to 6 pm). The acute intervention groups received one one-hour treatment session only. The NC and DC groups received no exercise or hypoxia intervention (they placed in the tent with normal air for the same time as the intervention groups).

Every effort was made to minimize animal pain, suffering, and distress. No unexpected mortality or adverse events were observed.

### 2.3 Variables measured

#### 2.3.1 Blood sampling for testing FBG

Blood glucose levels were measured using a hand-held blood glucose meter (SANNUO, China).

For the pre-intervention test, approximately 1 μL blood sample was taken from the saphenous vein after six hours fasting [36]. The fasting started at 7:00 am and there was no intervention was given during the fasting period).

For the acute effect, on the experimental day, the blood sample was collected after the fasting period, including one hour intervention (a total of 6 hour fasting), at 30 min post the intervention. For the chronic effects of IHI, the blood and tissues samples were collected 72 hours after the last intervention session (including the six hours fasting). The sampling time of long-term intervention is different from that of acute intervention, in order avoid the impact of stress response generated by 1-hour intervention on skeletal muscle indexes, which can reflect the adaptive changes produced by skeletal muscle [37, 38].

For both the acute and chronic effects, the post-intervention blood samples were collected when the animal was anaesthetized by an intraperitoneal injection of sodium pentobarbitone at a dosage of 60 mg/kg body weight (blood was taken from the inferior vena cava after euthanasia), and 80 μL blood was withdrawn from the inferior vena cava, for the measurement of FBG level and other variables.

#### 2.3.2 Muscle sampling

For the acute effect, the skeletal muscle samples were collected after the blood sampling post the intervention. For the chronic effects of IHI, the skeletal muscle samples were collected 72 hours after the last intervention session. Skeletal muscle samples were obtained from the quadriceps femoris for all groups. After the blood sample was taken as described above, the muscle sample was dissected, frozen in liquid nitrogen, and stored at −80°C before analysis.

#### 2.3.3 Measured blood variables

The plasma membrane was separated from the quadriceps muscle as a sample for testing GLUT4 content [39], and then measured using ELISA method (JL2050, Jianglai Biotechnology Co., LTD.) [40]. A commercial kit (NAD-2-Y, Keming Biotechnology Co., Ltd.) was used to detect the concentration of NADH and NAD^+^. The glucose concentration in skeletal muscle was detected using a Glucose Assay kit (F006-1-1, Keming Biotechnology Co., Ltd.). The concentration of lactate in skeletal muscle was detected by using a Lactate Assay kit (A019-2-1, Keming Biotechnology Co., Ltd.). The concentration of pyruvate in skeletal muscle was detected by using a pyruvate Assay kit (A081-1-1, Keming Biotechnology Co., Ltd.). The procedures were performed in strict accordance with the kits’ instructions (S1 Appendix).

### 2.4 Statistical analysis

All experimental data were processed using SPSS statistical software (IBM SPSS version 25 for Windows). One-way analysis of variance (ANOVA) was performed to compare the group means. The mean and standard deviation (SD) are presented for all variables measured. If a significant effect was detected, Bonferroni adjustment was used in post-hoc comparisons. The Shapiro-Wilk test was used to assess the data for a normal distribution, and Levene’s test was used to test the homogeneity of the variance. The data that were not normally distributed (glucose, NAD^+^, and NADH/NAD^+^ ratio) underwent logarithmic transformation before further analysis. The non-parametric Kruskal-Wallis H test was used for the data that remained not normally distributed after the logarithmic transformation (pyruvate and lactate/pyruvate ratio).

## 3 Results

### 3.1 FBG

**Fig 1:**
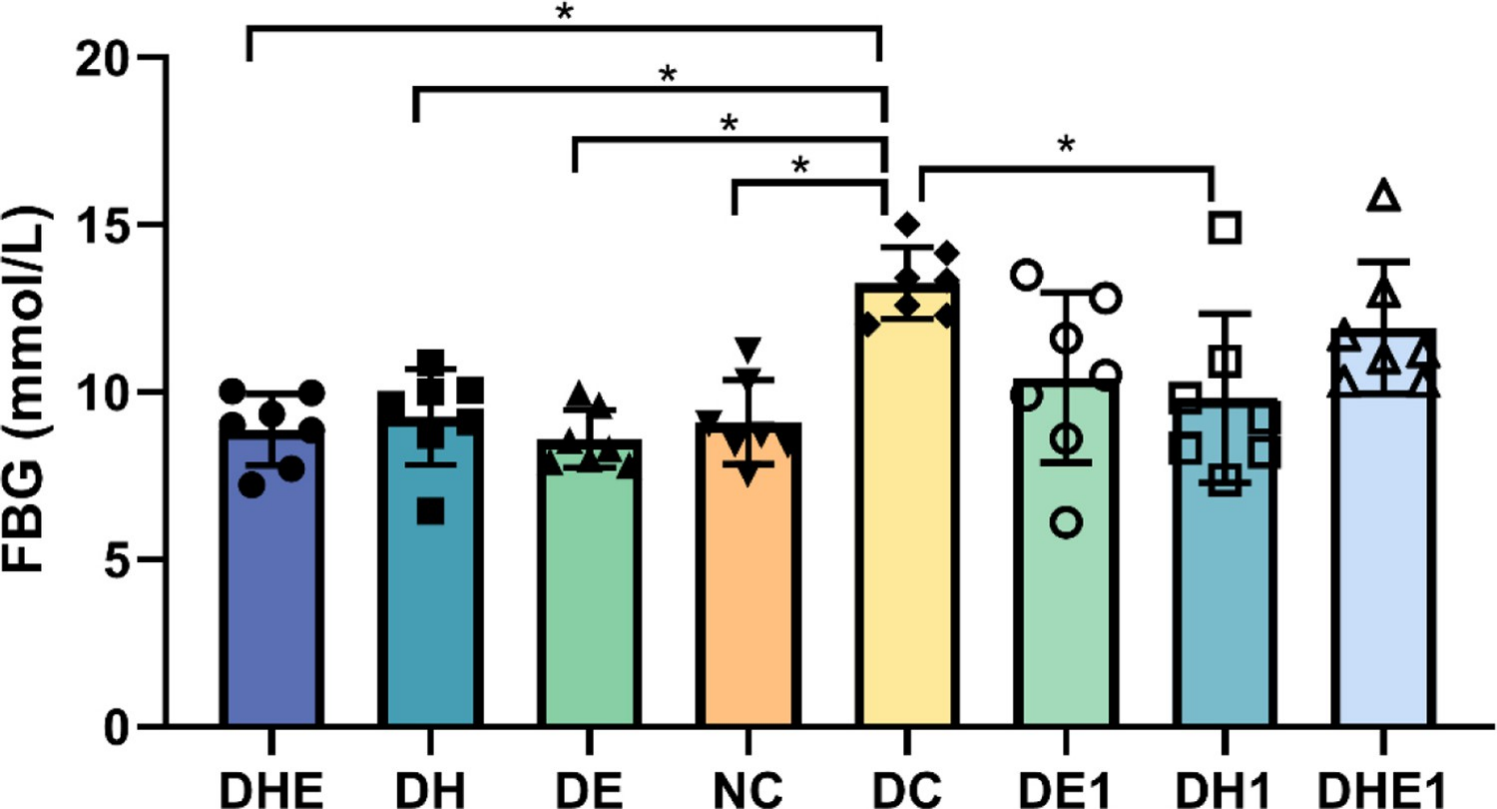
Fasting blood glucose (FBG) in skeletal muscle after the intervention. The error bars represent SD. * p < 0.05. NC represents control mice, sedentary in normoxic environment; DC, diabetic control mice, sedentary in normoxic environment; DE, diabetic mice, 4 weeks of exercise in normoxic environment; DH, diabetic mice, sedentary for 4 weeks in hypoxic environment; and DHE, diabetic mice, 4 weeks of exercise in hypoxic environment; DE1, diabetic mice, one hour exposure to exercise in normoxic environment; DH1, diabetic mice, one hour exposure to exercise in hypoxic environment; and DHE1, diabetic mice, one hour exposure to exercise in hypoxic environment. N = 7 in each group.

The results of one-way ANOVA indicated that there were differences in fasting blood glucose levels among the groups (F = 6.47, P < 0.000). Post-hoc comparisons indicated that fasting blood glucose levels in the DC were significantly higher than that in the NC group, all the four-week intervention groups (DH 9.26 ± 1.43 mmol/L,95% confidence intervals (CI) 7.94–10.58, DE 8.60 ± 0.86 mmol/L, 95% CI 7.80–9.39 and DHE 8.87 ± 1.07 mmol/L, 95% CI 7.88–9.86, P < 0.05), and the DH1 group (9.82 ± 2.53 mmol/L, 95% CI 7.48–12.16) (Fig 1).

### 3.2 GLUT4

**Fig 2:**
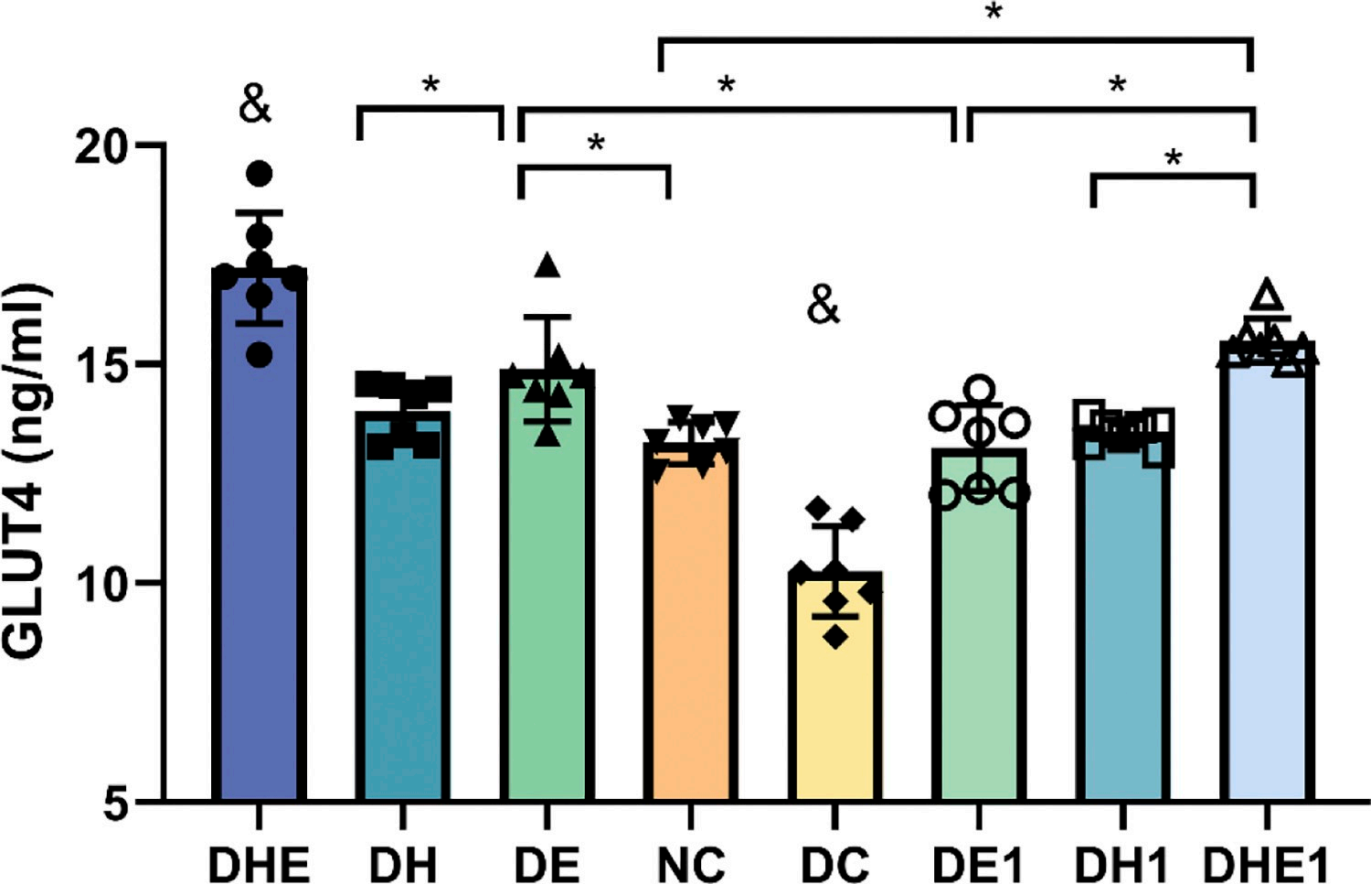
Expression of GLUT4 in muscle plasma membrane and after the intervention. The error bars represent SD. * p < 0.05. & Indicates that there is a significant difference as compared with all other groups. NC represents control mice, sedentary in normoxic environment; DC, diabetic control mice, sedentary in normoxic environment; DE, diabetic mice, 4 weeks of exercise in normoxic environment; DH, diabetic mice, sedentary for 4 weeks in hypoxic environment; and DHE, diabetic mice, 4 weeks of exercise in hypoxic environment; DE1, diabetic mice, one hour exposure to exercise in normoxic environment; DH1, diabetic mice, one hour exposure to exercise in hypoxic environment; and DHE1, diabetic mice, one hour exposure to exercise in hypoxic environment. N = 7 in each group.

The results of one-way ANOVA indicated that there were differences in the GLUT4 level between the groups (F = 39.560, P < 0.000). Post-hoc comparisons indicated that GLUT4 level in the DC (10.27 ± 1.03 ng/ml, 95% CI 9.32–11.22) were significantly lower than all other groups (P < 0.001). The level of GLUT4 protein in DHE (17.19 ± 1.27 ng/ml, 95% CI 16.02–18.37) was significantly higher than all other groups (P < 0.05). The GLUT4 level in the DHE1 (15.54 ± 0.50 ng/ml, 95% CI15.08–16.00) was significantly higher than that in the DE1 (13.90 ± 0.98 ng/ml, 95% CI 12.18–14.00), DH1 (13.45 ± 0.30 ng/ml 95% CI 13.18–13.73), DH (13.91 ± 0.67 ng/ml, 95% CI 13.30–14.53) and NC (13.2 ± 0.48 ng/ml, 95% CI 12.76–13.64) groups (P < 0.05). There were differences in GLUT4 between the DE (14.88 ± 1.20 ng/ml, 95% CI:13.78–15.99) and DE1 group (P = 0.01) (Fig 2).

### 3.3 Skeletal muscle glucose metabolites

#### 3.3.1 Glucose

**Fig 3:**
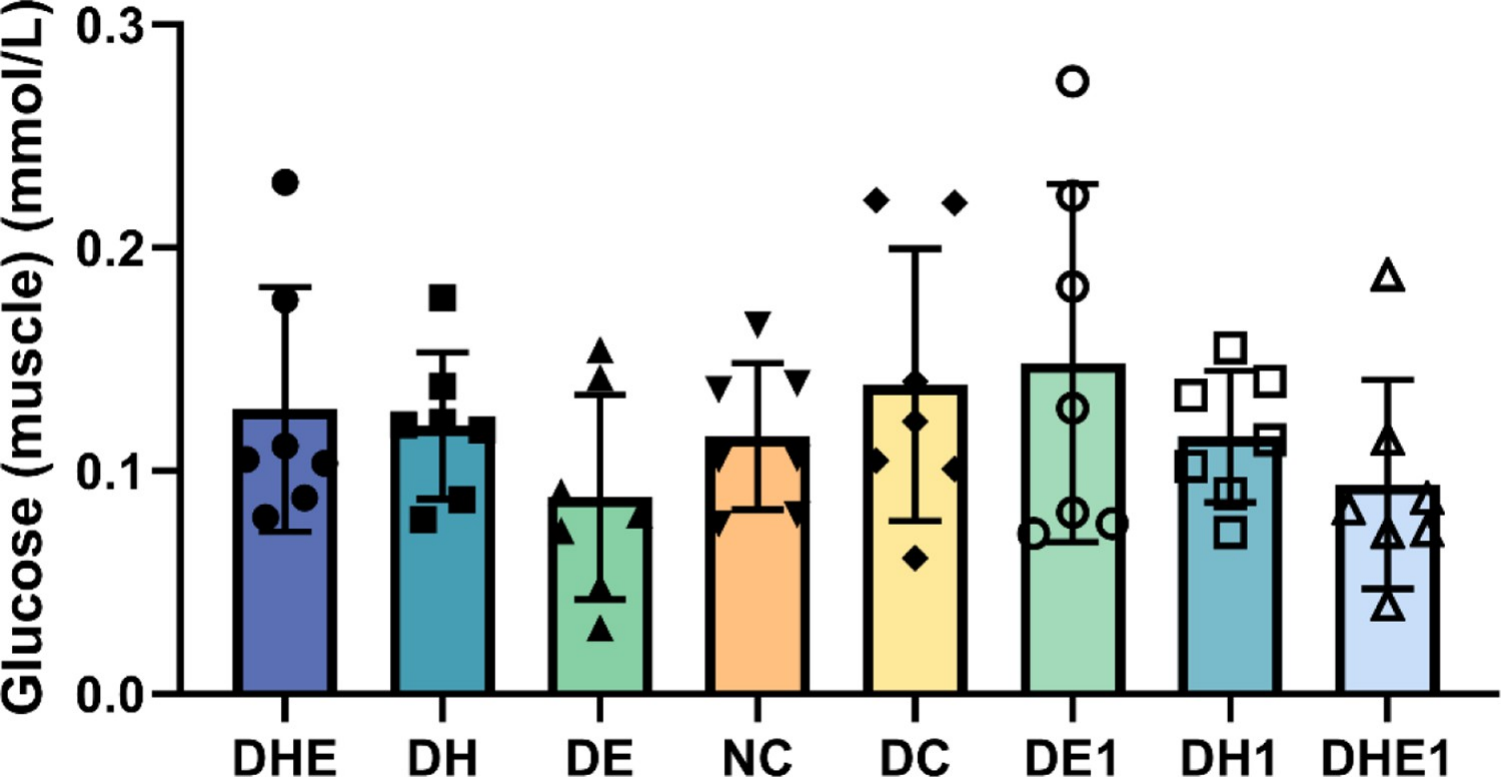
Muscle glucose concentration after the intervention. The error bars represent SD. NC represents control mice, sedentary in normoxic environment; DC, diabetic control mice, sedentary in normoxic environment; DE, diabetic mice, 4 weeks of exercise in normoxic environment; DH, diabetic mice, sedentary for 4 weeks in hypoxic environment; and DHE, diabetic mice, 4 weeks of exercise in hypoxic environment; DE1, diabetic mice, one hour exposure to exercise in normoxic environment; DH1, diabetic mice, one hour exposure to exercise in hypoxic environment; and DHE1, diabetic mice, one hour exposure to exercise in hypoxic environment. N = 7 in each group.

The results of the one-way ANOVA indicated that that there were no significant between-group differences in the concentrations of glucose in the muscle (F = 1.810, P = 0.107) (Fig 3).

#### 3.3.2 Lactate, pyruvate and the ratio of lactate/pyruvate

**Fig 4:**
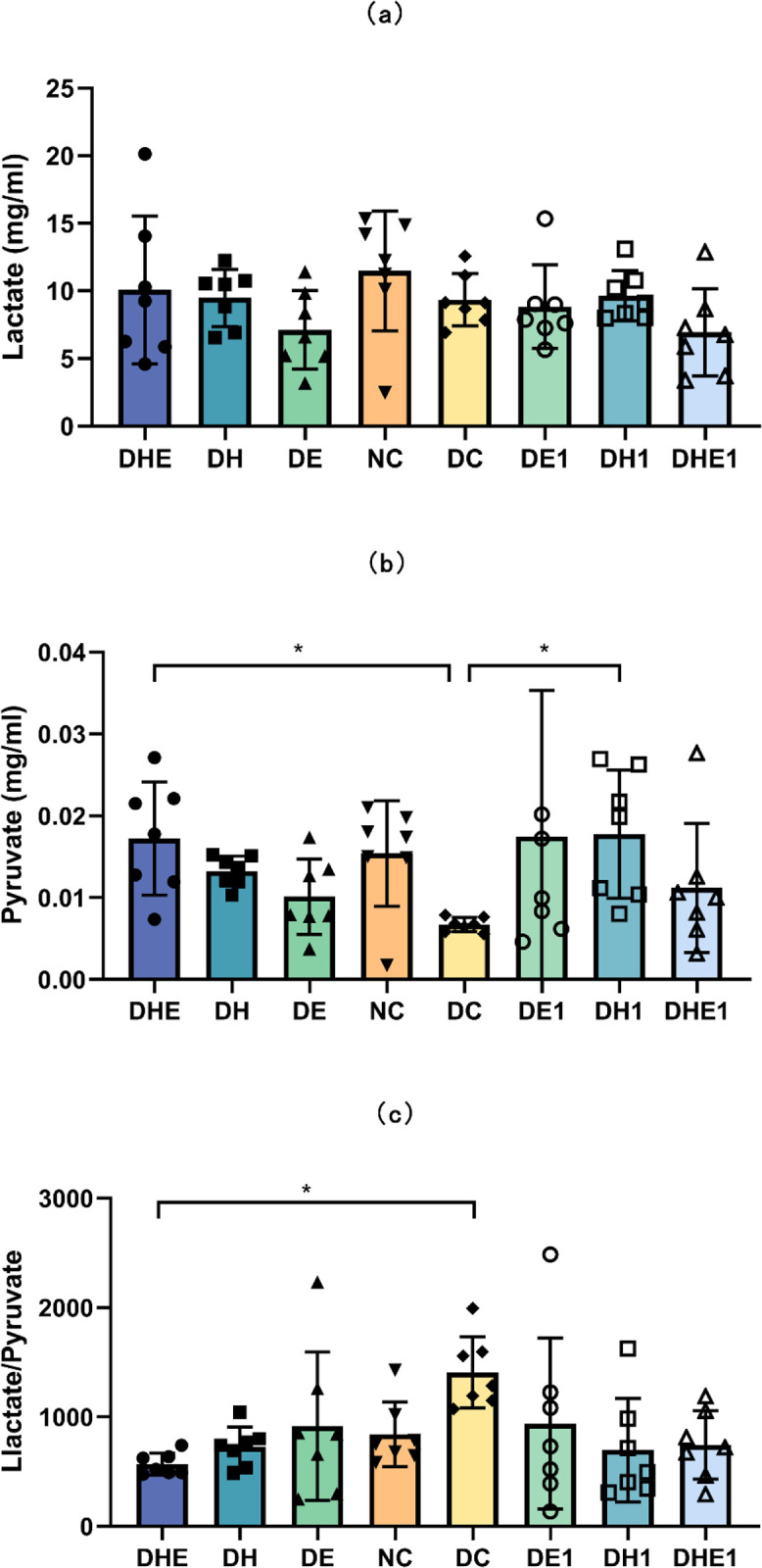
Lactate (a) and Pyruvate (b) concentrations and the ratio of lactate/pyruvate (c) in skeletal muscle after the intervention. The error bars represent SD. * p < 0.05. NC represents control mice, sedentary in normoxic environment; DC, diabetic control mice, sedentary in normoxic environment; DE, diabetic mice, 4 weeks of exercise in normoxic environment; DH, diabetic mice, sedentary for 4 weeks in hypoxic environment; and DHE, diabetic mice, 4 weeks of exercise in hypoxic environment; DE1, diabetic mice, one hour exposure to exercise in normoxic environment; DH1, diabetic mice, one hour exposure to exercise in hypoxic environment; and DHE1, diabetic mice, one hour exposure to exercise in hypoxic environment. N = 7 in each group.

The results of the one-way ANOVA indicated that there were no significant between-group differences in the concentration of lactate in the muscle (F = 1.413, P = 0.222) (Fig 4A).

The Kruskal-Wallis H test indicated that there was a significant difference in the pyruvate concentration (P = 0.012) (Fig 4B) and the ratio of lactate/pyruvate (P = 0.03) (Fig 4C). The pyruvate concentration of the DHE (0.017 ± 0.007 mg/ml, 95% CI 0.01–0.02) and DH1 (0.018 ± 0.008 mg/ml, 95% CI 0.01–0.03) were higher than that of the DC (0.007 ± 0.0008 mg/ml, 95% CI 0.006–0.008) group (P = 0.031). The ratio of lactate/pyruvate of the DHE (568.83 ± 100.57, 95% CI 475.82–661.85) was higher than that of the DC (1407.14 ± 325.53, 95% CI 1105.14–1709.13) group (P = 0.009).

#### 3.3.3 NADH, NAD^+^ and the ratio of NADH/NAD^+^

**Fig 5:**
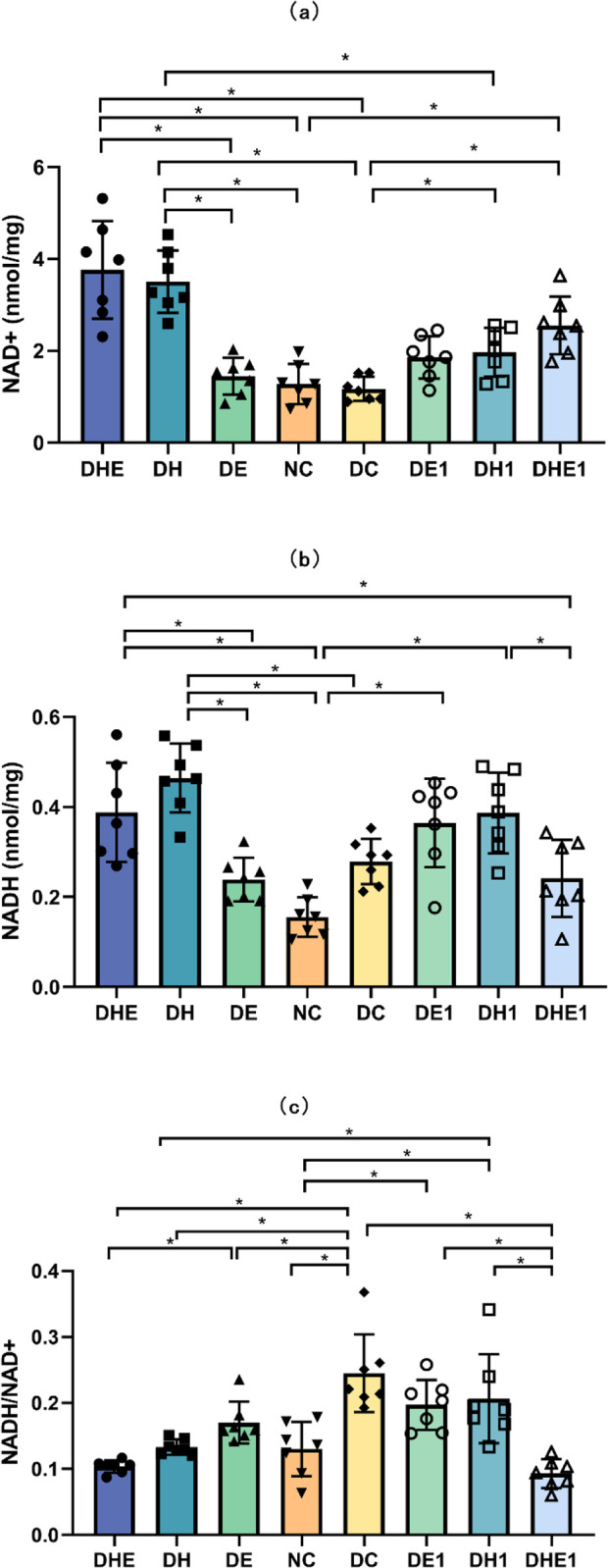
NAD^+^ (a) and NADH (b) concentrations, and the ratio of NADH/NAD^+^ (c) in skeletal muscle after the intervention. The error bars represent SD. * p < 0.05. NC represents control mice, sedentary in normoxic environment; DC, diabetic control mice, sedentary in normoxic environment; DE, diabetic mice, 4 weeks of exercise in normoxic environment; DH, diabetic mice, sedentary for 4 weeks in hypoxic environment; and DHE, diabetic mice, 4 weeks of exercise in hypoxic environment; DE1, diabetic mice, one hour exposure to exercise in normoxic environment; DH1, diabetic mice, one hour exposure to exercise in hypoxic environment; and DHE1, diabetic mice, one hour exposure to exercise in hypoxic environment. N = 7 in each group.

The results of the one-way ANOVA indicated that there was a significant difference in the concentration of NAD^+^ between the groups (F = 18.747, P < 0.001). Post-hoc comparisons indicated that the NAD^+^ concentration levels in the DHE (3.76 ± 1.06 nmol/mg, 95% CI 2.78–4.75) and DH (3.51 ± 0.68 nmol/mg, 95% CI 1.48–2.46) groups were significantly higher than NC (1.28 ± 0.44 nmol/mg, 95% CI 0.87–1.68), DC (1.17 ± 0.26 nmol/mg, 95% CI 0.93–1.41), and DE (1.45 ± 0.40 nmol/mg, 95% CI 1.07–1.81) groups (P < 0.001). The NAD^+^ in the DHE1 (2.55 ± 0.63 nmol/mg, 95% CI 1.97–3.14) was higher than NC and DC groups (P < 0.001); and the NAD^+^ in the DH1 (1.97 ± 0.53 nmol/mg, 95% CI 1.48–2.46) was also higher than DC (P = 0.031) groups (Fig 5A).

The results of the one-way ANOVA indicated that there was a significant difference in the concentration of NADH between the groups (F = 11.762, P < 0.001). Post-hoc comparisons indicated that the NADH in the DHE (0.39 ± 0.11 nmol/mg, 95% CI 0.29–0.49) was higher than DE (0.24 ± 0.05 nmol/mg, 95% CI 0.19–0.28, P = 0.026) and NC (0.16 ± 0.04 nmol/mg, 95% CI 0.11–0.20), P < 0.001) groups; and the NADH in the DH (0.46 ± 0.08 nmol/mg, 95% CI 0.39–0.54) was higher than DE, NC (P < 0.001) and DC group (0.28 ± 0.05 nmol/mg, 95% CI 0.23–0.33, P = 0.002). The NADH in the DH1 (0.39 ± 0.09 nmol/mg, 95% CI 0.34–0.47) was higher than NC (P < 0.001) and DHE1 (0.24 ± 0.09 nmol/mg, 95% CI 0.16–0.32, P = 0.033) groups, and the NADH in the DHE was significantly higher than that in the DHE1 group (P = 0.031) (Fig 5B).

The results of the one-way ANOVA indicated that there were significant differences in the ratio of NADH/NAD^+^ between the groups (F = 14.966, P < 0.001). The NADH/NAD^+^ ratio in the DC (0.25 ± 0.06, 95% CI 0.19–0.30) was significantly higher than that in the NC (0.13 ± 0.14, 95% CI 0.09–0.17, P < 0.001), DE (0.17 ± 0.03, 95% CI 0.14–0.20, P = 0.046), DH (0.13 ± 0.01, 95% CI 0.12–0.14, P < 0.001) and DHE (0.10 ± 0.01, 95% CI 0.09–0.11, P <0.001) groups. Post-hoc comparisons indicated that the ratio of NADH/NAD^+^ in the DHE1 (0.09 ± 0.02, 95% CI:0.07–0.11) was lower than that in the DC (P < 0.001), DE1 (0.20 ± 0.04, 95% CI 0.16–0.23, P < 0.001) and DH1 (0.21 ± 0.07, 95% CI 0.14–0.27, P < 0.001) groups. The ratio of NADH/NAD^+^ in the NC was lower than DC (P < 0.001), DE1 (P = 0.027) and DH1 (P = 0.011) group. The NADH/NAD^+^ ratio in the DH1 was significantly higher than that in the DH (P = 0.029) (Fig 5C).

## 4 Discussion

The results suggest an increased uptake and utilization of glucose in skeletal muscle after IHI. These findings suggest that the glycolysis and NAD cycle were promoted in the skeletal muscle and that this promotion may have contributed to the effects of the IHI, exercise, or a combination of both interventions in reducing blood glucose levels. In other words, these findings suggest that a readjustment of the balance between substrate availability and utilization occurred in the muscle. Therefore, the evidence from this study has provided new insight into the potential physiological mechanism underlying the effect of IHI on improvement of blood glucose management in the mouse model of T2D.

### 4.1 Skeletal muscle glucose metabolites after the 4 weeks of intervention

There may be a mismatch between the levels of availability and utilization of glucose in skeletal muscle under various health conditions [41]. Impaired insulin-stimulated glucose uptake by skeletal muscle has been reported in T2D rats [42]. Our findings are in line with these previous reports. Numerous studies have demonstrated the promotion of glucose uptake by increased GLUT4 translocation [43], which contribute to glycemic control [44]. Our results on blood glucose levels support this point (Fig 1). Exercising in a normoxic or hypoxic environments, or resting in a hypoxic environment, can lead to increased levels of GLUT4 translocation therefore would increase blood glucose uptake by the skeletal muscle. The measurement of glucose levels in skeletal muscle can be used to evaluate the dynamics of glucose metabolism in the cell [45]. However, the glucose level in the skeletal muscle of diabetic mice was not higher after the interventions (Fig 3). This may indicate that the increased available glucose might be quickly consumed in the skeletal muscle.

Glycolysis and aerobic oxidation in skeletal muscle tissue are the main pathways of glucose consumption [15]. Our results indicated that the interventions increased the level of glycolysis in the muscle of T2D mice, which is consistent with the results obtained by Petersen and Shulman [46]. The process of glycolysis breaks down one molecule of glucose to form two molecules of pyruvate [47]. Interestingly, only the DHE group showed a significant change in the glycolytic flux of the skeletal muscle in this mouse model of T2D (Fig 4B, 4C). Perhaps there is a synergy for glycolysis between exercise and hypoxia. The pyruvate produced in glycolysis has two fates. One fate is the participation in anaerobic glycolysis to produce lactate. The other fate is to enter the tricarboxylic acid cycle within the mitochondrial matrix to participate in the oxidative phosphorylation [48]. Therefore, the ratio of lactate/pyruvate actually reflects the balance between anaerobic and aerobic metabolism in the tissue [49]. The ratio of lactate/pyruvate in the DHE group was lower than that in the DC group (Fig 4C), suggesting that exercise in a hypoxic environment may have alleviated the defect of oxidative metabolism in T2D skeletal muscle.

Our results also indicated that the IHI (either alone or combined with exercise) reduced the NADH/NAD^+^ ratio more effectively than exercise alone. In the cells that exhibit insulin resistance, the TCA cycle metabolism is disordered [50–53] with an increased NADH level. It has been suggested that high levels of NADH influx in mitochondria have an inhibitory effect on enzymes in the TCA cycle [52] and reduce the substrate utilization rate. In our study, muscle NADH concentrations were higher in T2D mice under hypoxic conditions (DHE and DH), but the ratio of NADH/NAD^+^ was lower compared with the DC mice, suggesting that NADH was relatively decreased (Fig 5C). In addition, the concentrations of NAD^+^ in the DHE group were much higher than those in the DC group (Fig 5A), whereas the DE group did not show a significant change, indicating the effect of the hypoxia intervention. This is consistent with existing research results [54]. Previous studies have shown that a damage to the electron transport chain will hinder the mitochondrial NAD cycle (NADH consumption and NAD^+^ production), leading to NADH accumulation [52]. These obstacles are related to the development of T2D [55]. We found that the hypoxia intervention reduced NADH accumulation (i.e., no difference from the NC group) (Fig 5C). NAD is an important cofactor in metabolic process and mitochondrial adaptation [56]. Grange et al. reported that a hypoxia intervention increased NAD^+^ levels in mice with mitochondrial dysfunction [57]. Recent studies have also shown that an increased intracellular NAD+ levels can reduce stress and drive metabolic responses [58]. Therefore, IHI may increase the use of substrates by skeletal muscle, which may be due to the improvement by hypoxia on the electron transport chain [23].

### 4.2 Skeletal muscle glucose metabolites in response to the one-hour treatment

With respect to the acute responses following the exposure to one bout of hypoxia and or exercise, the DH1 and DHE1 groups had showed no change in the expression of GLUT4 in skeletal muscle (Fig 1). Notably, GLUT4 levels were higher in the DHE and DH groups compared with DHE1 and DH1 (Fig 2). This result suggests that the long-term IHI had an effect in enhancing the ability of skeletal muscle to uptake glucose. Exercise (chronic) has a cumulative effect on the effects of skeletal muscle glucose transport. Compared with T2D mice exposed to one bout of hypoxia (DH1), T2D mice with 4 weeks of hypoxia (DH) showed improved skeletal muscle NAD cycle (Fig 5). Therefore, it appears that the stimulation of one-hour exposure to IHI or exercise may not cause strong enough changes in the other variables measured in this study. Long-term intervention would allow the skeletal muscle to adapt to the intensity and frequency of IHI and enhance the glucose metabolism in skeletal muscle. In our study, there was a significant difference between acute stress and long-term adaptation in the body’s mitochondrial NAD cycle.

## 5 Conclusion

In summary, the results of this study suggests that the IHI can enhances the expression of GLUT4, a key protein of skeletal muscle glucose, and increases glycolysis while reducing the NADH/NAD^+^ ratio in skeletal muscle in a mouse model of diet-induced diabetes. The results also suggests that the IHI promotes both glucose uptake and utilization in T2D. A synergistic effect of exercise and hypoxia was observed in this study only at the level of glycolysis. In future studies, the synergistic effect of exercise and hypoxia on the improvement of mitochondrial morphology and mitochondrial function should be considered to examine the underlying mechanism of the IHI effects and to provide more comprehensive evidence to explain the effect of IHI on blood glucose in type 2 diabetes.

## Supporting information

S1 Appendix Measured blood variables. (DOCX)

S2 Appendix The data used in statistical analysis of the study. (XLSX)
