# Supplementary material for: Enhanced glucose utilization of skeletal muscle after 4 weeks of intermittent hypoxia in a mouse model of type 2 diabetes
Source: PLoS One. 2024 Jan 25;19(1):e0296815. doi: 10.1371/journal.pone.0296815 (PMC10810429; doi:10.1371/journal.pone.0296815)
Supplement: S1 Appendix — (DOCX) [file pone.0296815.s001.docx]

**Supporting information (S1)**

**1．Measured variables**

**1.1 Isolation of plasma membrane from quadriceps muscle for testing GLUT4**

Muscle samples are rinsed in Solution A (250 mmol/L Sucrose, 50 mmol/L Tris, 0.2 mmol/L EDTA, pH7.4) to remove fat, blood vessels and connective tissue. Centrifuge 120 g twice at 4°C for 15 min. The mixed supernatant was collected and centrifuged at 4℃ at 9000 g for 20 min. The supernatant was collected and centrifuged at 4℃ at 190,000 g for 60 min. The resulting precipitate is re-suspended in 2 mL solution A. The suspension was centrifuged in 25% sucrose solution at 4°C and 150,000 g for 16 hours. 25% sucrose layer was collected and washed with 20 mmol/L Tris-HCl solution diluted 4 times. The mixture was centrifuged at 190,000 g at 4°C for 60 min. The resulting particles were re-suspended with 100 μL of solution A as a sample (Klip et al., 1987). GLUT4 content was detected with the kit (JL2050, Jianglai Biotechnology Co., LTD.) (Schnurr et al., 2015).

**1.2 NADH and NAD^+^**

A commercial kit (NAD-2-Y, Keming Biotechnology Co., Ltd.) was used to detect the concentration of NADH and NAD^+^. The NADH and NAD^+^ in the sample were extracted with alkaline and acidic extracting solutions, respectively. NADH transfers hydrogen through N-methylphenazine methyl sulfate to reduce oxidized thiazole blue (thiazolyl blue tetrazolium bromide, often called MTT) to formazan. The concentration of formazan was detected using a spectrophotometer (NANO 2000, Thermo Fisher Scientific, U.S.A.) at an absorbance wavelength of 570 nm, as an indication of the NADH concentration. Alcohol dehydrogenase was used to reduce the NAD^+^ extracted from the sample to NADH, which was detected using the MTT reduction method.

One of two 15-mg muscle samples from each mouse was treated with an acid extraction solution (300 μL) for NAD^+^ and the other sample with an alkaline extraction solution for NADH and then homogenized in an ice bath. The homogenates were placed in a water bath at 90 °C for 5 min, and then cooled in an ice bath. The cooled sample was centrifuged at 4 ℃ with 10,000 *g* for 10 min. The supernatant was transferred to a new centrifuge tube, and equal volumes of an alkaline (for NAD^+^) or acidic (for NADH) extraction solution were added and mixed to neutralize the preparation. The sample was centrifuged again at 4 °C with 10,000 *g* for 10 min. The supernatant was obtained for testing.

A spectrophotometer was used to compare the absorbance at a wavelength of 570 nm with that of the control (tube A1, with reagents added according to the kit instructions) for concentrations of NADH and NAD^+^ (A2). The concentrations of NAD^+^ and NADH in the sample were calculated using the following formula:

$$\mathbf{NAD=}\frac{\boldsymbol{(\triangle A- 0.099 )\times36.1\times V1}}{\boldsymbol{V1\times[protein]}}$$

$$\mathbf{NADH=}\frac{\boldsymbol{(\triangle A- 0.065 )\times24.7\times V1}}{\boldsymbol{V1\times[protein]}}$$

Note: △A = A2−A1; A1: NADH absorbance value; A2: NAD^+^ absorbance value;

V1: sample volume in the reaction system; [protein]: protein concentration of the sample to be tested determined by the Coomassie brilliant blue method.

**1.3 Glucose**

The glucose concentration in skeletal muscle was detected using a Glucose Assay kit (F006-1-1, Keming Biotechnology Co., Ltd.) (Li et al., 2022; Yang et al., 2023). Muscle samples (50 mg) were added to normal saline (0.9% NaCl, 450 mL), homogenized in an ice bath, and centrifuged for 10 min at 4 ℃ and 1000 g. The supernatant (100 μL) was used to determine the cellular glucose concentration ([glucose]). The glucose in the sample was oxidized by glucose oxidase to generate hydrogen peroxide and gluconic acid. Hydrogen peroxide condenses phenols to quinones, which can be detected by spectrophotometry under the action of peroxidase.

We followed the kit instructions to determine the optical density (OD) values of the sample and the standard at a wavelength of 505 nm using a spectrophotometer. The assay was performed after adding the test solution or standard solution (5.55 mmol/L), a working solution, and distilled water to the sample or standard, and heating the samples in a water bath at 37 ℃ for 5 min. The glucose content (absolute quantification) was calculated using the following formula:

$$\mathbf{Glucose}\mathbf{=}\frac{\mathbf{sample OD value}}{\mathbf{standard OD value}}\boldsymbol{\times standard concentration\div[glucose]}$$

Note: Standard concentration = 5.55 mmol/L = 99.9 mg/dL; [glucose]: concentration of glucose determined using the Coomassie brilliant blue method.

**1.4 Lactate**

The concentration of lactate in skeletal muscle was detected by using a Lactate Assay kit (A019-2-1, Keming Biotechnology Co., Ltd.) (Zou et al., 2022; Wang et al., 2023a; Wang et al., 2023b). NAD^+^ was used as the hydrogen acceptor to convert NAD^+^ to NADH in the process of converting lactate to pyruvate by lactate dehydrogenase. N-methylphenazine methyl sulfate reduced nitrogen blue tetrazolium to a blue-purple substance, which showed a linear relationship with the lactate content at a wavelength of 530 nm. We diluted 10% of the homogenate to 2.5% (because our pre-test showed that 2.5% of the homogenate was within the best linear determination range of this kit), and 100 μL of the homogenate was used to determine the lactate concentration by the Coomassie blue staining method. According to the kit instructions, double-distilled water, a standard solution with a concentration of 3 mmol/L enzyme working solution, a chromogenic agent, and a termination solution were added to the standard tube and to the sample tube. The preparation was mixed using a scroll oscillator (Qilinbeier, Vortex-5). The OD values of the sample, the blank, and the standard were determined using a spectrophotometer (530 nm, 1 cm light path, double steaming water zeroing). The lactate content was calculated using the following formula：

$$\mathbf{Lact}\mathbf{ate}\mathbf{=}\frac{\mathbf{OD1-OD2}}{\mathbf{OD3-OD2}}\boldsymbol{\times C\div[lact}\mathbf{ate}\mathbf{]}$$

Note：C: standard concentration = 3 mmol/L; OD1: OD value of the sample; OD2: OD value of the blank; OD3: OD value of the standard; [lactate]: lactate concentration of the sample to be tested determined by the Coomassie brilliant blue method.

**1.5 Pyruvate**

The concentration of pyruvate in skeletal muscle was detected by using a Pyruvate Assay kit (A081-1-1, Keming Biotechnology Co., Ltd.) (Ma et al., 2020; Shen et al., 2022; Yang et al., 2023). The principle of the method is similar to that of lactate in that pyruvate will show as reddish-brown in an alkaline solution after reacting with a chromogenic agent (2,4-dinitrophenylhydrazine), and the intensity of the colour is proportional to the concentration of pyruvate. The OD value at a wavelength of 505 nm represented the content of pyruvate in the sample.

Double-distilled water, a pyruvate standard (0.2 μmol/mL), the tissue homogenate (10%), and a chromogenic agent were added to the standard and to the sample according to the kit instructions, and the solutions were mixed well. The mixtures stood for 5 min and then the OD values of sample, blank, and standard were determined by spectrophotometry at a wavelength of 505 nm. The pyruvate content was calculated using the following formula:

$$\mathbf{Pyr}\mathbf{u}\mathbf{v}\mathbf{ate}\mathbf{=}\frac{\mathbf{OD1-OD2}}{\mathbf{OD3-OD2}}\boldsymbol{\times}\mathbf{C}\boldsymbol{\div}\mathbf{[pyruv}\mathbf{ate}\mathbf{]}$$

Note: C: standard concentration = 3 mmol/L; OD1: optical density value of the sample; OD2, OD value of the blank; OD3, OD value of the standard; [pyruvate]: pyruvate concentration of the sample to be tested determined by the Coomassie brilliant blue method.

**2 Statistical analysis**

**Body weight**

The results of one-way ANOVA indicated that there were differences in the body weight between the groups (F = 3.647, P = 0.003). Post-hoc comparisons indicated that body weight in the DC group (38.66 ± 2.30g, 95% CI 33.03-44.28) was significantly higher than in the NC group (27.36 ± 2.21, 95% CI 21.95-32.78, p = 0.016). The groups of the DH1 (37.89 ± 2.58, 95% CI 31.58-44.20, p = 0.034), DE1 (38.49 ± 1.63, 95% CI 34.52-42.47, p = 0.019) and DHE1 groups (38.11 ± 1.75, 95% CI 33.83-42.39, p = 0.027) were significantly higher than that of the NC group (Fig 6).


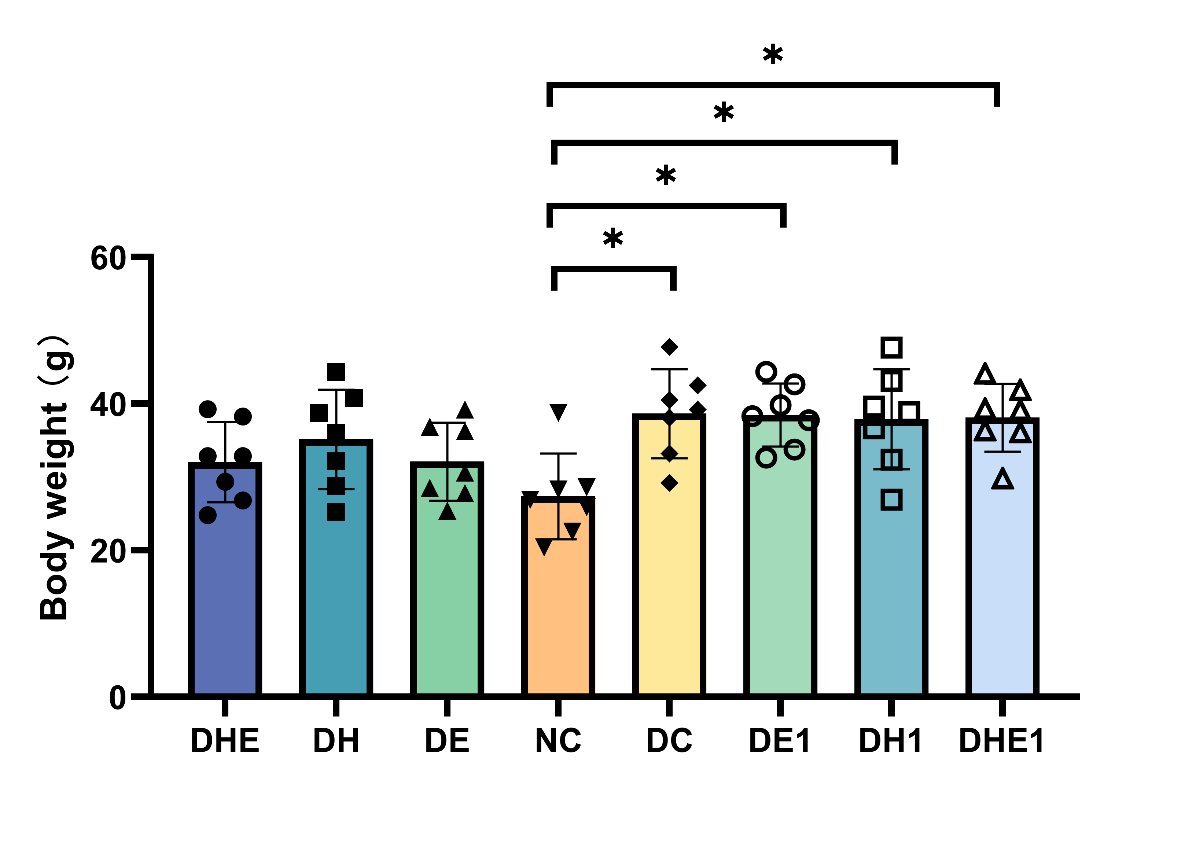


**Fig 6**. Effects of four weeks IHI intervention and one hour exposure to hypoxic environment on body weight. The error bars represent SD. * p < 0.05. NC represents control mice, sedentary in normoxic environment; DC, diabetic control mice, sedentary in normoxic environment; DE, diabetic mice, 4 weeks of exercise in normoxic environment; DH, diabetic mice, sedentary for 4 weeks in hypoxic environment; and DHE, diabetic mice, 4 weeks of exercise in hypoxic environment; DE1, diabetic mice, one hour exposure to exercise in normoxic environment; DH1, diabetic mice, one hour exposure to exercise in hypoxic environment; and DHE1, diabetic mice, one hour exposure to exercise in hypoxic environment. N = 7 in each group.

**References**

Klip, A., Ramlal, T., Young, D.A., and Holloszy, J.O. (1987). Insulin-induced translocation of glucose transporters in rat hindlimb muscles. *FEBS Lett* 224(1)**,** 224-230. doi: 10.1016/0014-5793(87)80452-0.

Li, H., Xi, Y., Liu, H., and Xin, X. (2022). Gypenosides ameliorate high-fat diet-induced non-alcoholic steatohepatitis via farnesoid X receptor activation. *Front Nutr* 9**,** 914079. doi: 10.3389/fnut.2022.914079.

Ma, M., Ma, C., Li, P., Ma, C., Ping, F., Li, W., et al. (2020). Low glucose enhanced metformin's inhibitory effect on pancreatic cancer cells by suppressing glycolysis and inducing energy stress via up-regulation of miR-210-5p. *Cell Cycle* 19(17)**,** 2168-2181. doi: 10.1080/15384101.2020.1796036.

Schnurr, T.M., Reynolds, A.J., Komac, A.M., Duffy, L.K., and Dunlap, K.L. (2015). The effect of acute exercise on GLUT4 levels in peripheral blood mononuclear cells of sled dogs. *Biochem Biophys Rep* 2**,** 45-49. doi: 10.1016/j.bbrep.2015.05.002.

Shen, L., Ma, J., Zhou, H., Chen, L., Tang, J., Zhang, K., et al. (2022). Plasma Metabolomic Profiling Reveals Preliminary Biomarkers of Pork Quality Based on pH Value. *Foods* 11(24). doi: 10.3390/foods11244005.

Wang, H., Liang, W., Wang, X., Zhan, Y., Wang, W., Yang, L., et al. (2023a). Notch mediates the glycolytic switch via PI3K/Akt signaling to support embryonic development. *Cell Mol Biol Lett* 28(1)**,** 50. doi: 10.1186/s11658-023-00459-4.

Wang, J.X., Qiao, F., Zhang, M.L., Chen, L.Q., Du, Z.Y., and Luo, Y. (2023b). Double-edged effect of sodium citrate in Nile tilapia (Oreochromis niloticus): Promoting lipid and protein deposition vs. causing hyperglycemia and insulin resistance. *Anim Nutr* 14**,** 303-314. doi: 10.1016/j.aninu.2023.06.005.

Yang, M., Pan, M., Huang, D., Liu, J., Guo, Y., Liu, Y., et al. (2023). Glucagon Promotes Gluconeogenesis through the GCGR/PKA/CREB/PGC-1alpha Pathway in Hepatocytes of the Japanese Flounder Paralichthys olivaceus. *Cells* 12(7). doi: 10.3390/cells12071098.

Zou, X., Yang, Y., Lin, F., Chen, J., Zhang, H., Li, L., et al. (2022). Lactate facilitates classical swine fever virus replication by enhancing cholesterol biosynthesis. *iScience* 25(11)**,** 105353. doi: 10.1016/j.isci.2022.105353.
